# *Notes from the Field:* Intestinal Colonization and Possible Iatrogenic Botulism in Mouse Bioassay–Negative Serum Specimens — Los Angeles County, California, November 2017

**DOI:** 10.15585/mmwr.mm6743a6

**Published:** 2018-11-02

**Authors:** Umme-Aiman Halai, Dawn Terashita, Moon Kim, Nicole Green, Suzanne R. Kalb, Kevin Chatham-Stephens, Sharon Balter

**Affiliations:** ^1^Los Angeles County Department of Public Health, California; ^2^National Center for Environmental Health, CDC; ^3^National Center for Emerging and Zoonotic Infectious Diseases, CDC.

Mouse bioassay (MBA) is the standard test for botulinum neurotoxin detection. Matrix-assisted laser desorption/ionization time-of-flight mass spectrometry (MALDI-TOF MS) can be 10–100 times more sensitive than MBA (*1*), but is not yet widely available. This report describes two patients whose serum initially tested negative for botulinum neurotoxin by MBA and subsequently tested positive by MALDI-TOF MS. Los Angeles County Department of Public Health (LACDPH) routinely sends botulism test specimens to the CDC for MALDI-TOF MS while performing MBA in-house.

## Case 1

In mid-November 2017, an elderly man with no serious medical problems was admitted to a hospital with dysarthria, dysphagia, and dyspnea of 3 days’ duration. The day after admission (day 4 after symptom onset), he required endotracheal intubation and mechanical ventilation for respiratory failure. He developed ptosis, extraocular palsy, and quadriparesis. On day 16 (13 days into his hospitalization) LACDPH was consulted regarding suspected botulism and promptly released heptavalent botulinum antitoxin (HBAT). Clinicians subsequently decided not to administer HBAT because they suspected that refractory myasthenia gravis was the more likely diagnosis. A limited electromyography was nondiagnostic. Serum collected on day 16 was reported 8 days later to be negative for botulinum neurotoxin by MBA.

Interviews and home inspection did not identify high-risk foods or ill contacts, and the patient did not have any known risk factors for botulism, such as anatomic or functional bowel abnormalities or altered gastrointestinal flora associated with receipt of recent antimicrobials. The patient’s neurologic status did not improve, and MALDI-TOF MS results, available on day 35 (19 days after serum collection), confirmed botulinum toxin type A. HBAT treatment was discussed after this result, and a clinical decision was made to hold off HBAT administration. Stool collected on day 48 was positive for botulinum neurotoxin type A by MBA on day 68. HBAT was subsequently administered within 24 hours of this result (day 69), but neurologic status remained unchanged. The patient died from ventilator-associated pneumonia on day 109. The prolonged excretion of toxin-producing *Clostridium botulinum* is consistent with adult intestinal colonization botulism. Only one to two cases of adult intestinal colonization botulism are identified in the United States annually (*2*).

## Case 2

In late November 2017, a middle-aged woman was evaluated at a hospital emergency department with dysphagia and dysphonia without respiratory distress or weakness. Five days earlier, she had received botulinum toxin A injections using Food and Drug Administration (FDA)-approved doses for cervical dystonia at office A under electromyography guidance. The emergency department physician consulted LACDPH regarding suspected iatrogenic botulism; LACDPH released antitoxin, which the patient received. The next day, her symptoms had improved, and she was discharged from the hospital. Serum collected the day of symptom onset, before HBAT administration, tested negative for botulinum neurotoxin by MBA (on day 5); however, MALDI-TOF MS results available on day 41 confirmed botulinum neurotoxin type A. Office A reported the adverse event* to FDA’s MedWatch. Inspection of office A did not identify injection practice or dosing concerns. It is unknown whether the patient’s dysphagia and dysphonia, which are localized effects expected from botulinum toxin administration, would have progressed to systemic signs and symptoms had HBAT not been administered.

Laboratory results for these patients highlight the reported increased sensitivity of MALDI-TOF MS compared with MBA, with potential implications for botulism surveillance. Because MALDI-TOF MS testing is not available locally at LACDPH, specimens must be shipped to CDC for testing, and results might not be available until 2–4 weeks later. Because paralysis can develop rapidly, HBAT should be administered empirically for most suspected botulism cases on the basis of clinical findings (*3*) to prevent progression or respiratory failure. Clinicians are required to report suspected cases of botulism immediately to local and state public health officials who are available around-the-clock for consultation, release of antitoxin, exposure identification, and guidance on laboratory testing and interpretation. For a patient with localized signs and symptoms after botulinum toxin injection, clinicians, in close consultation with public health officials, might consider monitoring the patient and administering HBAT if additional signs or symptoms of neurologic weakness suggesting systemic spread of toxin occur.

## Footnotes

* Detection of botulinum toxin in serum after an FDA-approved botulinum toxin formulation administered at an approved dose for an approved indication.
